# Spectral analysis assisted photoacoustic imaging for lipid composition differentiation

**DOI:** 10.1016/j.pacs.2017.05.002

**Published:** 2017-06-06

**Authors:** Yingchun Cao, Ayeeshik Kole, Lu Lan, Pu Wang, Jie Hui, Michael Sturek, Ji-Xin Cheng

**Affiliations:** a Weldon School of Biomedical Engineering, Purdue University, West Lafayette, IN 47907, USA; b Department of Cellular & Integrative Physiology, Indiana University School of Medicine, Indianapolis, IN 46202, USA; c Department of Physics and Astronomy, Purdue University, West Lafayette, IN 47907, USA; d Department of Chemistry, Purdue University, West Lafayette, IN 47907, USA

**Keywords:** Photoacoustic imaging, Spectral analysis, *k*-means clustering, Lipid composition, Atherosclerosis

## Abstract

Recent advances in atherosclerotic plaque detection have shown that not only does lipid core size and depth play important roles in plaque rupture and thrombi formation, but lipid composition, especially cholesterol deposition, is equally important in determining lesion vulnerability. Here, we demonstrate a spectral analysis assisted photoacoustic imaging approach to differentiate and map lipid compositions within an artery wall. The approach is based on the classification of spectral curves obtained from the sliding windows along time-of-flight photoacoustic signals via a numerical *k*-means clustering method. The evaluation result on a vessel-mimicking phantom containing cholesterol and olive oil shows accuracy and efficiency of this method, suggesting the potential to apply this approach in assessment of atherosclerotic plaques.

## 1 Introduction

Cardiovascular disease has been the leading cause of death over the past century in developed countries [1]. Atherosclerosis is recognized as the pathologic basis of cardiovascular disease, in which lipids accumulate in an artery wall leading to plaque growth and subsequent obstructive lumen narrowing or rupture [2]. The vulnerable, or rupture-prone plaque is typically characterized by a thin fibrous cap, a large lipid-rich necrotic core, and inflammatory infiltrate [3], [4]. Of these hallmarks, lipid accumulation has been shown to be the most frequently observed precondition of a plaque rupture [5]. The majority of lipids present in an atherosclerotic plaque are free cholesterol and its esterified form [6]. Cholesterol found in early atherosclerotic lesions participates the formation of macrophage foam cells, while crystalline cholesterol is thought to induce plaque rupture by physical disruption of the fibrous cap [7]. Therefore, cholesterol crystals have been identified as an important hallmark of inflammation and atherosclerotic lesions [8], [9]. Cholesteryl esters mainly accumulate in cytoplasmic droplets [2] and constitute a major fraction of lipid-rich necrotic core [10]. Their abundance is highly associated with plaque rupture and thrombi formation [8]. Therefore, the differentiation of crystalline cholesterol from its esterified form in an intact artery is of particular importance for the histopathological classification of advanced atherosclerotic lesions, as well as early diagnostics of the burden of an artery [11].

The widely used methods for studying the cholesterol deposition in atherosclerotic plaques are histological stains. However, none of these methods can label both crystalline cholesterol and cholesteryl ester. Microscopic imaging modalities such as confocal laser reflection microscopy [9] and micro-optical coherence tomography [12] have been successfully applied to visualize reflective components, but neither can provide chemical information of the target. Spontaneous [13] and coherent Raman scattering [14] have been implemented for cholesterol imaging with high performance. However, these approaches are limited as they are not compatible for *in vivo* imaging of an intact artery.

Catheter-based imaging modalities, such as intravascular ultrasound (IVUS) [15], optical coherence tomography (OCT) [16], and near-infrared spectroscopy (NIRS) [17], have been developed to interrogate the artery structure from inside the vessel. IVUS can show the artery morphology with high quality [17]. By combining with virtual histology, it can map the composition distribution within the plaque, thus classify the plaque type [17], [18]. However, the accuracy of virtual histology IVUS has been challenged, especially for the accurate quantification of necrotic core size. [19], [20]. OCT provides excellent spatial resolution of the artery, but it can neither penetrate sufficient depth nor provide compositional information. NIRS is only able to differentiate arterial composition in a very thin layer; thus, not providing depth resolution.

Intravascular photoacoustic (IVPA) imaging is an emerging technique converting optical absorption into ultrasonic wave and detecting it with a sensitive transducer integrated in the same catheter probe through intravascular implementation [21], [22]. This imaging technique and catheter design allows for concurrent co-registered IVPA/US imaging. In IVPA systems, excitation wavelengths of 1.2 μm [23] or 1.7 μm [24], [25], corresponding to the second and first vibrational overtone absorptions of C—H bond [26], [27], were usually utilized to selectively map lipid deposition in atherosclerotic plaque. Although current systems can provide important information of the lipid core size, they lack the ability to separate crystalline cholesterol from cholesteryl ester, thus limiting the capability for accurate identification of the plaque vulnerability and inflammation. Spectroscopic IVPA imaging allows excellent differentiation of different lipid compositions [28]. However, this approach requires multiple wavelength scanning, which considerably sacrifices the imaging speed making it impractical for clinical applications.

Spectral parameters (e.g., slope, y-intercept and mid-band fit) of photoacoustic signals can be utilized to characterize and quantify different tissue types based on their microstructural and mechanical properties [29], [30], [31]. In this work, we applied spectral analysis of photoacoustic signals to IVPA imaging to differentiate and map lipid compositions. Different from existing approaches of spectral analysis in both ultrasound and photoacoustic imaging that are based on a set of spectral parameters, here we obtained the spectral curves for each sliding window along A-lines via fast Fourier transform. After calibration and normalization, the spectral curve sections within the effective frequency band of the transducer were selected for *k*-means clustering [32] to classify the chemical components. This method was demonstrated with imaging of a vessel-mimicking phantom composed by cholesterol and olive oil to validate its accuracy and feasibility in differentiating lipid compositions.

## 2 Methods

### 2.1 IVPA imaging system

**Fig. 1:**
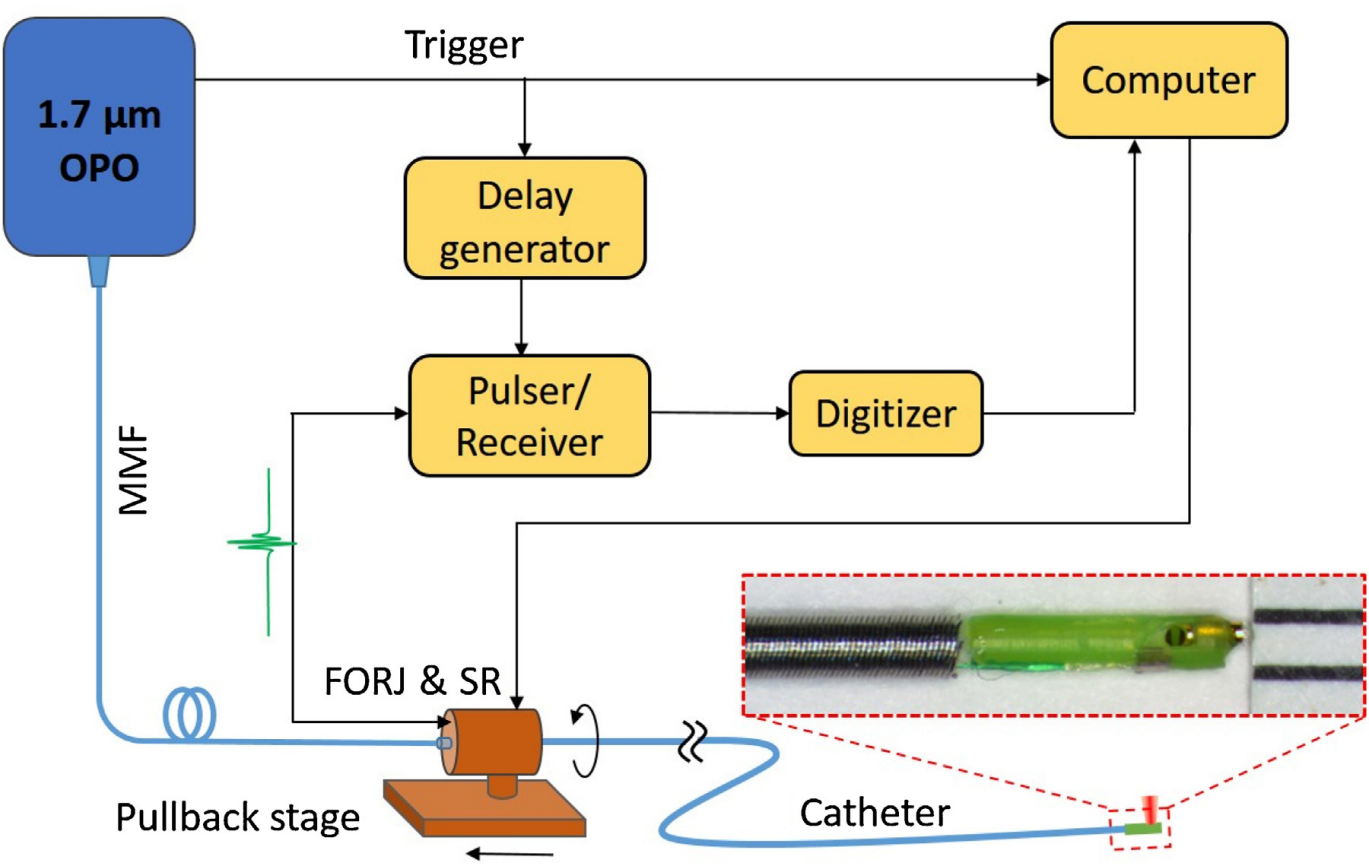
Schematic of the IVPA imaging system. The inset shows the photograph of IVPA catheter probe. OPO: optical parametric oscillator; MMF, multimode fiber; FORJ, fiber-optic rotary joint; SR, slip ring.

The IVPA imaging system used in this work is described in Fig. 1. A lab-built optical parametric oscillator (OPO) emitting at 1.7 μm with 500 Hz repetition rate [24] was used as the excitation source for photoacoustic generation. A section of multimode fiber coupled the light output from OPO to the IVPA catheter via a self-designed fiber-optic rotary joint (FORJ). The imaging catheter with a collinear optical/acoustic design and a diameter of 1 mm offers highly sensitive and co-registered ultrasound and photoacoustic imaging (Fig. 1, inset) [33]. The imbedded ultrasound transducer has a center frequency of 42 MHz and bandwidth of 50% (Blatek, Inc.). The FORJ, driven by a computer-controlled motor (SM17205D, Moog Inc.), was employed with a linear pullback stage to perform 3-D intravascular imaging. The photoacoustic signal was detected by an installed transducer in the probe and sent to a pulser/receiver (5073PR, Olympus, Inc.) with a 39-dB amplification factor through an electrical slip ring. Within a single optical pulse, a delayed ultrasound wave was sent and echo received by the same transducer via an OPO-triggered delay generator (9500+, Quantum Composers, Inc.) and pulser/receiver. A 16-bit digitizer (ATS9462, AlazerTech, Canada) with 180 MS/s sampling rate was used to collect the detected signals for further imaging reconstruction and analysis. The whole process was controlled by a LabView-based program. Optical pulse energy from the catheter tip was maintained at ∼100 μJ to below the 1.0 J/cm^2^ ANSI safety standard for skin at 1.7 μm [34]. For the experiment in this work, the FORJ was rotated at a speed of 1 revolution per second, which corresponds to an imaging speed of 1 frame per second and 500 A-lines for each cross-sectional image.

### 2.2 Procedure for photoacoustic spectral analysis

**Fig. 2:**
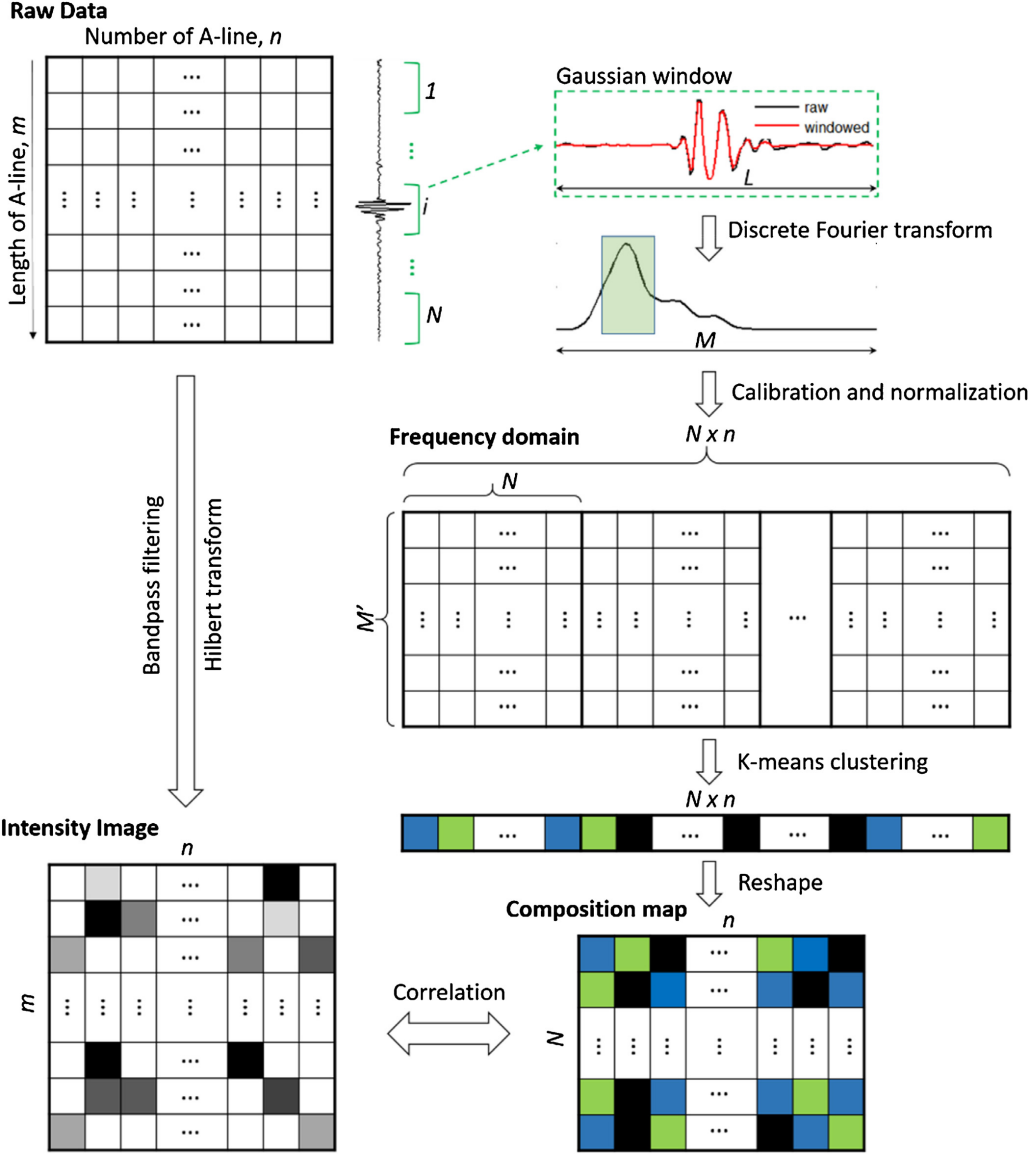
Numerical procedure for chemical composition differentiation by spectral analysis of photoacoustic signals. *m* and *n* denote the length of each A-line and total number of A-lines, *L* is the length of each Gaussian windows and *N* is the total Gaussian windows along an A-line, *M* denotes the length of Fourier transform of a Gaussian window and *M*’ represents the length of tailored spectrum of a Gaussian window.

Our method of differentiating lipid components in atherosclerotic plaque is based on spectral analysis and classification of photoacoustic signals via *k*-means clustering. The main procedure is sketched in Fig. 2. The raw data for a B-scanned image is first expressed as conventional intensity distribution of photoacoustic signal by bandpass filtering and Hilbert transform for each time-of-flight A-line. To perform spectral analysis, a sliding segment of raw data with fixed sliding step along each A-line is applied with a Gaussian window to remove spectral leakage (i.e., suppress spectral side lobes) [30], [35] and then converted into the frequency domain by discrete Fourier transform. Each sliding window will represent a pixel in the spectral analyzed image to be generated. The spectral curve of each selected window is calibrated by the sensitivity spectral curve of the transducer to remove the effect from transducer, and then normalized for the part falling into the bandwidth of the transducer prior generating a frequency domain data matrix to avoid the artifacts from intensity classification and to reduce the burden of calculation. Each row of the frequency domain matrix represents the entire pixels in the composition map to be generated, while each column shows tailored spectral curve for each sliding window. Next, the spectral matrix is classified by *k*-means clustering with expected number of compositions plus background, with each individual composition assigned with an integer starting from 1. The composition array is subsequently reshaped to form a composition map. The composition map is further resized by the pixels of intensity image for accurate correlation. For intravascular application, the intensity image and composition map will be further expressed in polar coordinate to reconstruct the real world object. All these procedures are implemented by a MATLAB program written in our lab.

### 2.3 Phantom design

**Fig. 3:**
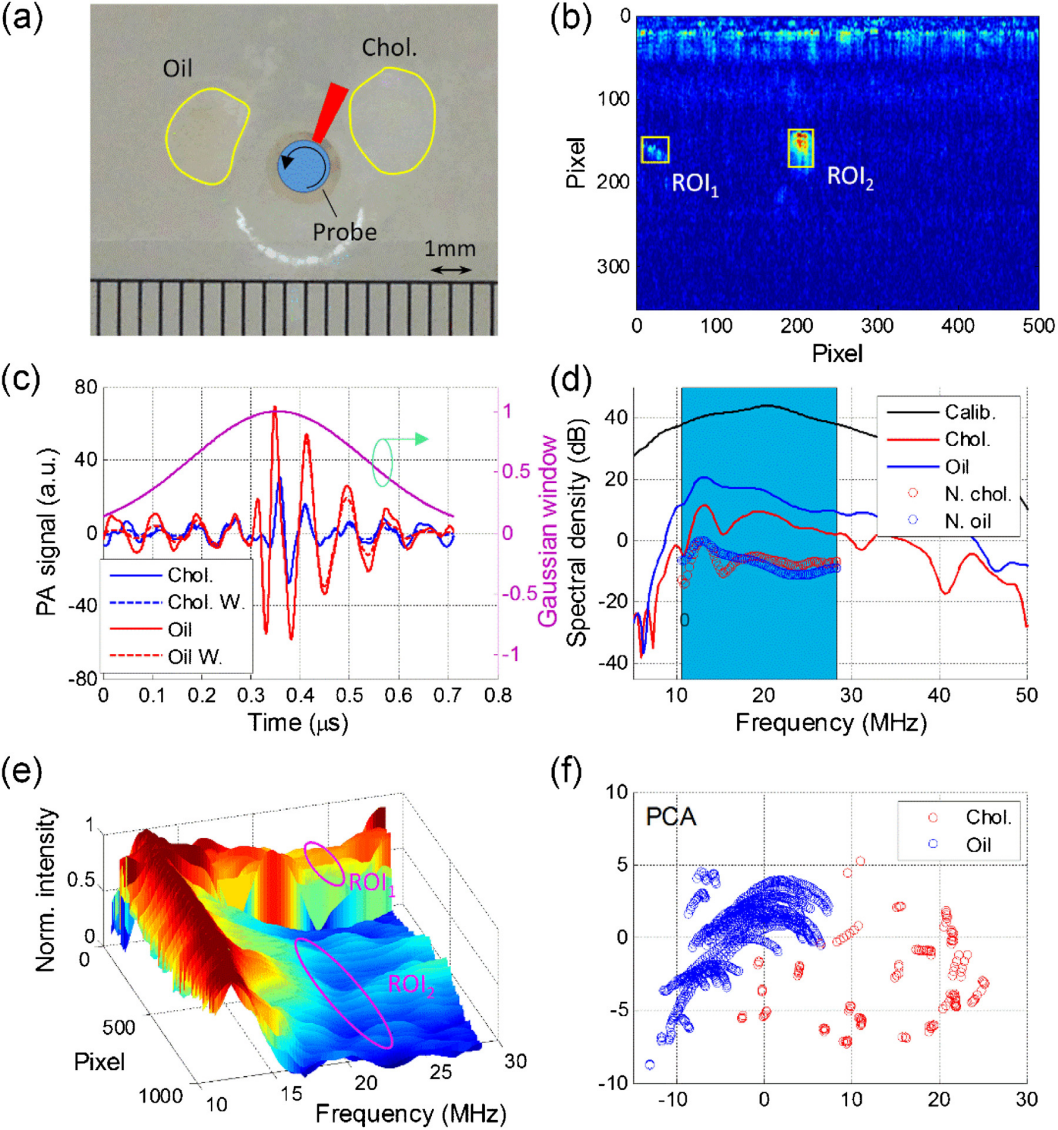
Spectral analysis of the two regions of interest. (a) A photograph of the phantom composed by cholesterol and olive oil, the central hole represents lumen of artery with catheter probe inserted in and rotational scanning for imaging; (b) Reconstructed A-line intensity distribution of the phantom with ROIs marked by yellow squares, ROI_1_ represents for cholesterol and ROI_2_ shows olive oil; (c) Photoacoustic signals within a Gaussian window at the cholesterol and olive oil positions; (d) Power spectra density in dB for signals shown in Fig. 3(c), the blue window from 10.5 to 28.3 MHz indicating the bandwidth of the transducer; (e) Normalized power spectral density within the bandwidth for ROI_1_ and ROI_2_; (f) PCA result for cholesterol and olive oil within the ROIs. Chol.: cholesterol; ROI: region of interest; W. and N.: windowed and normalized; PCA: principal component analysis.

A vessel-mimicking phantom with a cylindrical shape was prepared for IVPA imaging to verify the feasibility of our method. The medium of the phantom is composed by 1.5% (w/w) agarose gel with 1X phosphate buffered saline (PBS) to act as the tissue environment, with a 1.8-mm central hole reserved to mimic the lumen of the artery. Crystalline cholesterol (C8667, Sigma-Aldrich Co.) and olive oil (from market) were selected as the lipid compositions to represent the cholesterol crystal and esterified cholesterol, respectively, in atherosclerotic plaque considering their similar mechanical and chemical properties [36]. The shapes and locations of the compositions relative to the lumen can be visualized from Fig. 3(a).

## 3 Results

The phantom containing crystalline cholesterol and olive oil, with their boundaries outlined as in Fig. 3(a), was used for IVPA imaging. The reconstructed A-lines of photoacoustic signals shown in Fig. 3(b). However, one still cannot tell these two compositions from each other simply from the intensity distribution. Prior to applying *k*-means clustering, principle component analysis (PCA) was performed to the photoacoustic spectra of the target regions of interest (ROIs) containing these two compositions, as marked with yellow boxes in Fig. 3(b), to test the feasibility of differentiation this two compositions by the frequency response of their photoacoustic signal [37]. The peak photoacoustic signals within these two ROIs were selected with a Gaussian window with length of 129 pixels (or 0.72 μs) and standard deviation of 32 pixels along the A-line. To increase the accuracy of the Fourier transform, an interpolation level of 10 was applied to the raw A-line segments and their windowed signals as shown in Fig. 3(c). After windowed, their spectral curves, with a length of 4097 points determined by the window length in this work, at the peak values for both ROIs were plotted in Fig. 3(d). To avoid the influence from non-uniform sensitivity of the transducer, these two frequency curves were calibrated by the spectral curve of the transducer obtained by the echo from a stainless steel block. The calibrated frequency spectra were further tailored to the effective band range of the transducer (10.5-28.3 MHz, 6 dB from the peak of the spectral curve of the transducer) to reduce the calculation burden and improve accuracy later, and then normalized to unit (0 dB here) to avoid the misclassification from signal magnitude difference. It is shown in Fig. 3(d) that the spectral profiles of cholesterol and olive oil are very distinctive. We repeated this process over the entire ROIs and displayed their spectral curves in a 3-D plot as in Fig. 3(e), where we observe two distinctive groups of spectral profiles at these two ROIs. Subsequently, we applied PCA to these two regions to obtain the principle components of their spectra. The first two dimensions of PCA result, with eigenvalues weighted over 80% of all the components, was displayed in Fig. 3(f). The red and blue circles represent cholesterol and olive oil within the ROIs, respectively. It is shown that these two compositions are well separated from each other in the 2-D PCA distribution, indicating that they can be differentiated or clustered by proper method.

**Fig. 4:**
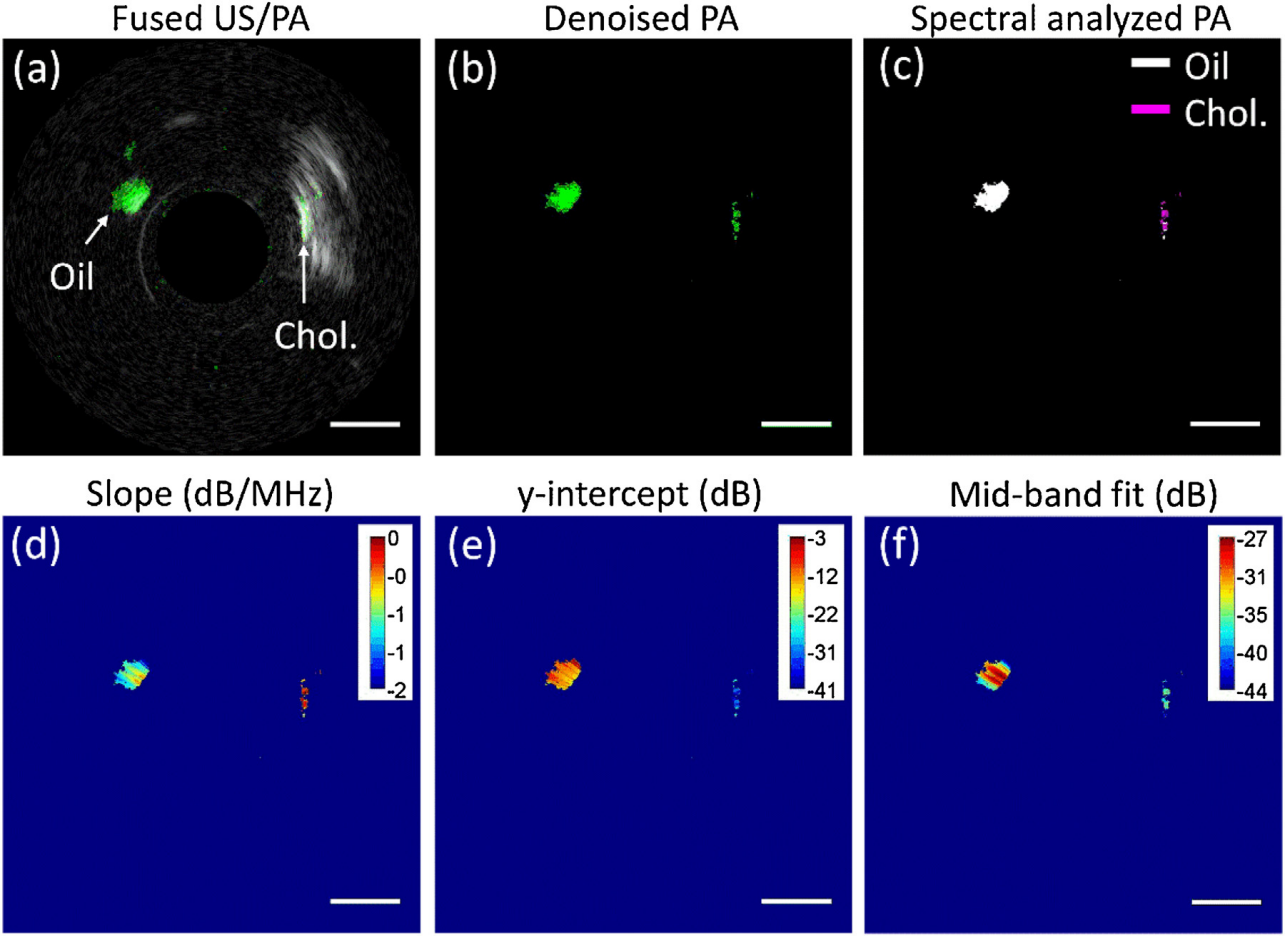
Photoacoustic image and derived composition map by spectral analysis. (a) Fused ultrasound/photoacoustic image with cholesterol and olive oil indicted by arrows; (b) Photoacoustic image by removing the signals below a background threshold; (c) Reconstructed composition map with cholesterol and oil marked by purple and white colors, respectively; (d)–(f) Spectral parameter maps for slope, y-intercept and mid-band fit, respectively. The 1 mm scale bar applies to all the panels.

The composition mapping is accomplished based on the aforementioned spectral analysis. The traditional ultrasound and photoacoustic intensity images of the phantom are fused and shown in Fig. 4(a), with cholesterol and olive oil indicated by arrows. It should be noted that olive oil did not show up in the ultrasound image due to the similar acoustic impedance of olive oil and agarose gel, while the area of detected cholesterol in the photoacoustic channel is much smaller than its actual size due to the strong light intensity loss in cholesterol crystal. For a more efficient calculation, signal intensity below a defined threshold (here set as 5σ_n_, where σ_n_ represents the background noise level) is excluded from the spectral analysis process. The photoacoustic image with noise removed is shown in Fig. 4(b). The pixels with effective photoacoustic signals were collected using a Gaussian window and their normalized spectra were obtained and arranged in sequence to form a frequency-domain matrix. Subsequently, *k*-means clustering was applied to these spectra over the entire image. Each cluster will be assigned with a *k*-means index starting from 1. The assigned *k*-means index array was reshaped and match to the dimension of the conventional photoacoustic image by pixel to generate a composition map as shown in Fig. 4(c). Here, cholesterol and olive oil are shown in purple and white colors, respectively. In order to evaluate the validity of this method, the accuracy of lipid differentiation is defined as the area of truly differentiated lipid components over the total imaged lipid area. According to the pre-designed spatial distribution of the lipid compositions, the accuracy of lipid differentiation in this phantom is measured to be as high as 98.4%.

To verify the results of our method, we also performed conventional spectral analysis of the photoacoustic image with noised removed by taking advantage of the developed slope, y-intercept and mid-band fit parameters method [18], [30]. That is the slope, y-intercept and mid-band of the calibrated spectra for each sliding window falling within the effective bandwidth of the transducer were obtained and reconstructed. The obtained maps for slope, y-intercept and mid-band fit distributions are displayed in Fig. 4(d)–(f) in polar coordinate to facilitate the comparison with real objective and our spectral analysis approach. It is found that the distributions of these three parameters for cholesterol and olive oil regions are very different as well, indicating distinct biochemical compositions in these two regions. They agree well with the results obtained by our *k*-means clustering method, further verifying the feasibility of our method for lipid composition differentiation.

## 4 Discussion

IVPA provides an exciting opportunity for lipid-specific imaging and detection. The co-registered IVPA/US imaging approach in this work has the advantages of IVUS on providing the morphologic information of the artery. Moreover, it offers added benefit of efficient chemically-selective detection of lipid in biological tissue. Through frequency spectral analysis of the photoacoustic signal, we showed that lipid compositions within the artery wall can be potentially differentiated and mapped with high spatial and chemical accuracy. This has not yet been proved to be possible with existing intravascular imaging approaches, such as virtual histology IVUS, which relies on the spectral parameter analysis of ultrasound echo to classify tissue types, and NIRS, which can only provide estimated possibility of lipid appearance in a specific direction, but no depth resolution. Therefore, identification of cholesterol concentration and lipid compositions is beyond their capabilities.

Although it has been demonstrated that lipid compositions within biological tissue can be differentiated by spectroscopic photoacoustic imaging [28], the necessity for laser wavelength scanning and data processing is not practical in a clinical setting. Moreover, multispectral photoacoustic imaging method sets much more requirements to the laser system, controlling and data processing, which are also not practical in the clinic.

Publications on photoacoustic spectral analysis showed an alternative for tissue characterization [30], [38]. They followed the method used in virtual histology IVUS and differentiated tissue types based on the distribution patterns of spectral parameters, such as slope, y-intercept and mid-band fit, within regions of interest. Although it can be helpful in some situations, this method is not able to distinguish tissue compositions and map their distributions.

The approach presented in this work demonstrated an efficient and accurate means for differentiation of lipid composition with several advantages: 1) a single laser wavelength is used; therefore, the imaging speed can be sufficient for the clinical setting; 2) the method is based on the analysis of raw IVPA signals, thus no extra hardware cost will be introduced; 3) through a k-means clustering approach, the same chemical components will be marked with an individual integer, i.e., individual color in the image, which is easy for users to understand; 4) the spectral curves have been calibrated and normalized before clustering; therefore, the errors introduced by different transducers, chemical concentrations will be reduced to the minimum; and 5) different from reported photoacoustic spectral analysis approaches, our method can generate a chemical distribution map over the entire image with spatial correspondence. Although we applied spectral analysis only to IVPA imaging in this paper, where an imaging depth of 5 mm will be sufficient enough to cover the entire artery, this method is also applicable to photoacoustic tomography for different species differentiation without laser wavelength scanning. The accuracy will be maintained considering the fact that the frequency of photoacoustic signal is determined by the mechanical property of the target.

## 5 Conclusions

In conclusion, we demonstrated a spectral analysis assisted IVPA approach for lipid composition differentiation, which can be potentially applied in atherosclerotic plaque detection and identification. This method is based on a spectral curve classification of each sliding window along the photoacoustic A-line signal through *k*-means clustering. The reconstructed composition map provides complementary chemical information to the typical photoacoustic/ultrasound image, which is valuable for the tissue type characterization and atherosclerotic plaque vulnerability identification.

## Conflict of interest

The authors declare that there is no conflict of interest. J.-X.C. and P.W. have a financial interested in Vibronix Inc., which, however, does not support this work.
